# Enhancing Mechanical Properties of Chitosan–Silica Aerogels with Tricalcium Phosphate Nanoparticles: A Molecular Dynamics Study for Bone Tissue Engineering

**DOI:** 10.3390/polym17060755

**Published:** 2025-03-13

**Authors:** Ali Attaeyan, Mohamad Shahgholi, Arash Karimipour

**Affiliations:** 1 Department of Mechanical Engineering, Najafabad Branch, Islamic Azad University, Najafabad 8514143131, Iran; 2 Department of Civil Engineering, Cihan University-Erbil, Erbil 44001, Iraq

**Keywords:** nanocomposite, chitosan, silica, tricalcium phosphate, molecular dynamic simulation

## Abstract

Chitosan–silica aerogel nanocomposites are lightweight materials with a highly porous structure that have a wide range of applications, including drug delivery systems, tissue engineering, and insulation. These materials may be strengthened using tricalcium phosphate in chitosan–silica aerogel nanocomposites. Thus, in the present research projects, the influence of different atomic percentages of TCP (2%, 3%, and 5%) on mechanical parameters such as stress-strain, ultimate strength, and Young’s modulus of chitosan–silica aerogel NCs was evaluated using molecular dynamics modeling and LAMMPS software. The findings demonstrate that the addition of tricalcium phosphate (1–3%) enhanced the ultimate strength and Young’s modulus of the simulated nanocomposite from 26.968 to 43.468 GPa and from 681.145 to 1053.183 MPa, respectively. The ultimate strength and Young’s modulus of the silica aerogel/chitosan nanocomposites, however, decreased to 1021.418 MPa and 42.008 GPa, respectively, with the addition more than 5% TCP.

## 1. Introduction

NCs are made up of two components: the matrix or base and nano-scale distributed fillers. These materials have attracted interest due to their unique features and potential applications in a number of scientific sectors and industry. However, in particular applications, the mechanical performance of NCs should be improved and strengthened to meet specific criteria [1,2,3,4]. TCP is a biocompatible and bioactive ceramic material that has been widely explored in a variety of sectors. It was discovered to improve the mechanical properties of several NCs. TCP is noteworthy for its biocompatibility and bioactivity, making it appropriate for medical and dental applications. TCP, which closely matches the mineral portion of bone, may be utilized as a bone replacement material. Furthermore, when implanted in the body, TCP may enhance bone formation and integrity, emphasizing its significance in orthopedic and dental procedures [5,6,7,8,9,10]. Regarding its potential advantages, TCP has been used as a reinforcing ingredient in a variety of composite materials. When added to certain materials, TCP serves as a reinforcing agent, increasing strength and hardness. Furthermore, the presence of TCP influences the microstructure and morphology of the NCs’ mechanical properties. Adding TCP particles changes the dispersion and distribution of fillers, which improves mechanical strength and toughness [11,12,13,14]. Chitosan–silica aerogel nanocomposites are lightweight materials distinguished by a highly porous architecture, making them suitable for diverse applications, such as tissue engineering, drug delivery systems, and insulation materials [15]. However, their mechanical properties, like tensile strength and elastic modulus, often need adjustment to satisfy particular needs. TCP in chitosan–silica aerogel nanocomposites serves as a reinforcing agent, leading to improved strength and hardness of these materials [16,17,18]. The addition of TCP particles strengthened the adhesion between the reinforcement and the chitosan–silica matrix, leading to better mechanical performance and higher load transfer. The mechanical strength of the material may be further improved by modifying the distribution of pore diameters and increasing overall density by the incorporation of TCP particles [19,20].

Moreno et al. [21] compared xerogels to aerogels and investigated the properties of chitosan–silica hybrid biomaterials for bone tissue engineering. In addition to chitosan and silica, the researchers examined adding 10% TCP by weight, which caused a quick bioactive reaction on the xerogel surface. Their findings reveal that xerogels stimulated cell differentiation at a quicker pace than aerogels with the same composition. As a result, the bioactive response of chitosan–silica xerogels and aerogels improved, as did their bone conduction and cell differentiation properties, because of sol-gel production of these materials. Shahi et al. [22] investigated the physical and mechanical properties of an NC scaffold constructed from β-TCP/PHB for bone tissue engineering applications. β-TCP and PHB-NPs were the main components of this NC scaffold. Their findings demonstrate that the NC scaffold had a highly porous structure with interconnected pores, which was very beneficial for nutrition transfer and cell penetration in tissue engineering applications. Adding β-TCP NPs improved the mechanical characteristics of the scaffold, including compressive strength and modulus, compared to pure PHB scaffolds. Moreno et al. [23] evaluated the impact of washing treatment on the tissue properties and bioactivity of silica/chitosan/TCP subgels for bone regeneration. They used a synthetic technique to create silica/chitosan/TCP xerogels with nominal amounts of silica chitosan (varying from 4% to 40% by weight) and TCP (ranging from 10% to 20% by weight). Their findings indicate that washing had a substantial effect on the bioactivity and textural properties of the silica/chitosan/TCP xerogels. Compared to untreated samples, treated samples showed increased specific surface area, pore structures, and porosity. Liu et al. [24] investigated how processing conditions influenced the microstructure and mechanical behavior of a silica–calcium phosphate NC. They found that increasing the sintering temperature (T) resulted in the production of a molten phase and greater particle size using XRD analysis. The mechanical characteristics of the NC were found to be dependent on the microstructure of the material and the amount of melt phase. The compressive strength and modulus of elasticity were similar to cortical bone, ranging from 62 to 204 MPa and from 6 to 14 GPa, respectively. Hu et al. [25] studied the mechanical properties of TCP-HA composite ceramics. Their findings indicated an optimal fracture hardness and toughness of 5 GPa and 1.27 MPa, respectively. These findings were superior to those obtained utilizing conventional mechanical mixing procedures. The increased mechanical characteristics were attributed to the efficient dispersion of the ACP phase and HA, as well as the crystallization of ACP.

TCP was particularly effective in enhancing the mechanical properties of chitosan–silica aerogel NCs. Currently, no quantitative studies have been undertaken to determine the influence of different atomic ratios of TCP NPs on the mechanical characteristics of chitosan–silica aerogel NCs. As a consequence, MD simulation and LAMMPS software were used to evaluate the influence of various atomic percentages of TCP (1%, 2%, 3%, and 5%) on mechanical parameters such as stress–strain, US, and YM of chitosan–silica aerogel NCs for bone tissue engineering applications. TCP percentages were determined using a mix of theoretical assumptions and available research. TCP is often utilized in bone tissue engineering to increase bioactivity and osteoconduction. For example, a research study on silica–chitosan xerogels included 10 wt.% TCP, which considerably enhanced pore volume and size, making it more suited for biomedical applications [21]. We used smaller percentages (1%, 2%, 3%, and 5%) to investigate the impacts of TCP at various concentrations, with the goal of optimizing mechanical qualities while maintaining bioactivity. This method enabled us to determine the ideal TCP concentration that balances mechanical strength and bioactive characteristics, which was critical for bone tissue engineering applications [26].

## 2. Simulation Approach

### 2.1. MD Simulation

The structural and dynamic properties of complex molecular systems, such as biomolecules and nanomaterials, can be investigated at the atomic scale using molecular simulations, which are a potent instrument. These simulations offer a comprehensive understanding of the behavior of atoms and molecules under a variety of conditions by numerically solving the equations of motion derived from classical mechanics. The models incorporate the interactions among particles, such as electrostatic interactions, covalent bonds, van der Waals forces, and other intermolecular forces, which enables the precise prediction of system behavior [27].

(1) $$F_{i}=m_{i}a_{i}=-\nabla_{i}U=-\frac{dU}{dr_{i}}$$

Computational methods are indispensable for simulating the complex behavior of large molecular systems. The velocity-Verlet algorithm is a widely used method for simulating atomic trajectories due to its accuracy. The algorithm facilitates the precise monitoring of particles’ movement in dynamic environments by employing Taylor series expansions and Newton’s laws of motion. It is an effective instrument for researchers who are pursuing detailed insights into molecular systems, as its capacity to incorporate higher-order terms enhances the accuracy of simulations [28,29,30].

(2) $$r_{i}\;\left(t+\Delta t\right)=2\;r_{i}\left(t\right)-r_{i}\left(t-\Delta t\right)+\left(\frac{d^{2}r_{i}}{dt^{2}}\right)\;{\left(\Delta t\right)}^{2}$$

(3) $$v\left(t+\Delta t\right)=v\left(t\right)+\Delta t\;v\left(t\right)+\frac{\Delta t\left(a\left(t\right)+a\left(t+\Delta t\right)\right)}{2}$$

The selection of potential functions is essential in MD simulations to ensure that the interactions between particles are faithfully represented. These functions specify the manner in which the particles influence one another in accordance with their relative positions and the categories of forces that are at play, including electrostatic interactions and van der Waals forces. The dynamics of the simulated environment are explained in terms of the total energy of the system, which is derived from these interactions.

(4) $$E_{total}=E_{bonded}+E_{nonbonded}$$

A comprehension of the forces that regulate atomic and molecular interactions is essential in MD simulations. A diverse array of potential functions is implemented to characterize the manner in which particles interact in accordance with their relative positions. The Lennard-Jones potential is one of the most frequently employed models, as it effectively balances both repulsive forces at short distances and attractive forces at extended distances [31]. These interactions are crucial for elucidating the behavior of gases, liquids, and solids, particularly during phase transitions and in molecular simulations of material properties. The Lennard-Jones potential’s simplicity and efficacy enable researchers to predict and analyze molecular behaviors in a variety of systems [32].

(5) $$U_{LJ}=4\varepsilon \left[{\left(\frac{\sigma }{r}\right)}^{12}-{\left(\frac{\sigma }{r}\right)}^{6}\right]\;r<r_{c}$$

Whereas σij denotes the distance at which the attractive interactions among particles become insignificant, εij which measures the intensity of these forces, shows the depth of a potential well. These characteristics, which are established via exact computations and experimental investigations, are essential for characterizing intermolecular interactions [33,34].

(6) $$\varepsilon_{ij}=\sqrt{\varepsilon_{i}\varepsilon_{j}}$$

(7) $$\sigma_{ij}=\frac{\sigma_{i}+\sigma_{j}}{2}$$

The parameters for each simulated element should be sourced from pertinent studies in the literature that are consistent with the physical concept of the simulated samples. In Table 1, these values are illustrated.

The Tersoff potential is a many-body potential that can be used to simulate materials, such as silicon and carbon [35]. It is capable of capturing the intricate dynamics that govern the interactions among atoms in these covalent systems by incorporating both repulsive and attractive forces. This framework is designed to facilitate the understanding of these interactions.

(8) $$E=\frac{1}{2}\;\underset{i}{\sum }\underset{j\neq i}{\sum }U_{ij}$$

(9) $$U_{ij}=f_{C}\left(r_{ij}\right)\left[a_{ij}f_{R}\left(r_{ij}\right)+b_{ij}f_{A}\left(r_{ij}\right)\right]$$

In this equation, the repulsive f_R_(r_ij_) and interactive f_A_(r_ij_) pair potentials, which govern atomic interactions, are determined by distinct equations.

This study also utilized the Vashishta potential function, an alternative interatomic potential model that incorporates many-body effects, in addition to simple pairwise interactions, to model silica aerogels. Unlike conventional models that only consider the distance between atom pairs, this approach accounts for variations in bond angles and atomic orientations, resulting in a more precise representation of atomic arrangements [36].

(10) $$U=\sum_{i}^{N}\sum_{j>i}^{N}U_{ij}^{\left(2\right)}(r_{ij})+\sum_{i}^{N}\sum_{j\neq i}^{N}\sum_{k>j,k\neq i}^{N}U_{ijk}^{\left(3\right)}(r_{ij},r_{ik},\theta_{ijk})$$

(11) $$U_{ij}^{\left(2\right)}(r)=\frac{H_{ij}}{r^{\eta_{ij}}}+\frac{Z_{i}Z_{j}}{r}\exp(\frac{-r}{\lambda_{1,ij}})-\frac{D_{\mathrm{ij}}}{r^{4}}\exp(\frac{-r}{\lambda_{4,ij}})-\frac{W_{ij}}{r^{6}},r<r_{c,ij}$$

(12) $$U_{ijk}^{\left(3\right)}(r_{ij},r_{ik},\theta_{ijk})=B_{ijk}\frac{{\left[\cos\theta_{ijk}-\cos\theta_{0ijk}\right]}^{2}}{1+C_{ijk}{\left[\cos\theta_{ijk}-\cos\theta_{0ijk}\right]}^{2}}\times \exp(\frac{\gamma_{ij}}{r_{\mathrm{ij}}-r_{0,ij}})\exp(\frac{\gamma_{ik}}{r_{\mathrm{ik}}-r_{0,ik}}),r_{ij}<r_{0,ij},r_{ik}<r_{0,ik}$$

### 2.2. Details of Simulation

**Figure 1:**
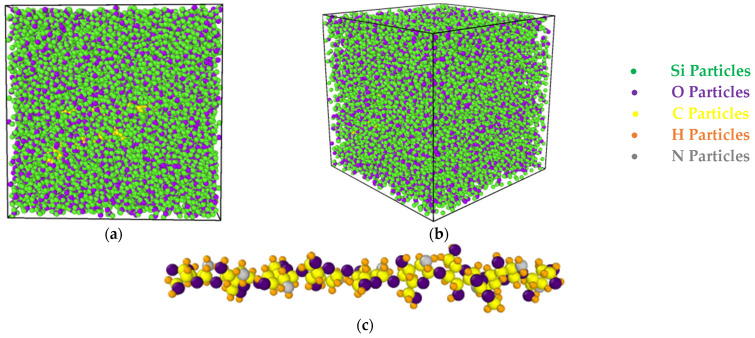
Atomic structure of chitosan/silica aerogel/TCP NC from (**a**) side and (**b**) perspective view and (**c**) chitosan nanostructure.

The preceding sections examined the role of TCP as a reinforcing agent in chitosan–silica aerogel NCs, which led to an increase in the strength and hardness of these materials. The effects of varying atomic percentages of TCP (2, 3, and 5%) in chitosan–silica aerogel NCs were examined in this study using MD simulations and LAMMPS (30 July 2021 version) software. The coordinates of atoms and their connections were included in the initial configuration. The simulation box dimensions for this investigation were established at 110 Å. The initial step entailed the modeling of silica aerogel using Avogadro software [37]. The NVT ensemble was employed to generate a sample at 7000 K for 1 ns in order to replicate the silica aerogel. The NVT ensemble was employed to gradually decrease T to 300 K over a period of 1 ns in the second stage. The structure with the NPT ensemble was minimized by adjusting T and pressure to 300 K and 1 bar for 1 ns in the third phase. The NVT ensemble was then employed to increase T to 3000 K over a 1 ns period. Ultimately, T decreased from 3000 K to 0 K. The chitosan and TCP nanostructures were subsequently extended in the simulation box using Packmol (V.20.16.0) software and Avogadro (V.1.95) software [38]. Figure 1 illustrates the atomic structure of chitosan/silica aerogel/TCP from various angles. The equilibrium of the structure was determined by the NVT ensemble after the initial modeling. A Nose–Hover thermostat was employed to equilibrate the necessary structure at an initial T of 300 K. This study investigated physical variables, including potential energy (PE). Subsequently, the ensemble transitioned to NVT, and the mechanical properties of the structure were examined.

### 2.3. Limitations

One of the most significant restrictions of molecular dynamics (MD) modeling is the tiny size of the simulation box, which limits the number of atoms that can be represented. This constraint may have an impact on the findings’ correctness, since many material characteristics in actual systems are dependent on long-range interactions and macroscopic behavior. Furthermore, the small system size may cause surface effects to be too prominent, resulting in differences between the simulated findings and the actual behavior of the material. To address this issue, periodic boundary conditions (PBCs) are used, which duplicate the simulation box in all directions. This method reduces finite-size effects and provides a more realistic picture of the material’s structural behavior on a greater scale. As a result, macroscopic characteristics may be examined by simulating nanoscale interactions, allowing for a more realistic analysis of the composite material under investigation

## 3. Results and Discussion

According to the information provided before, chitosan–silica aerogel NCs are lightweight materials with a wide range of applications, including tissue engineering, drug delivery systems, and insulation. TCP was used as a reinforcing agent in these NCs, increasing their mechanical characteristics, such as elastic modulus and tensile strength. Using MD simulations and LAMMPS software, this study investigated the impacts of different atomic percentages of TCP (2%, 3%, and 5%) in chitosan–silica aerogel NCs. This study was divided into two phases: first, the equilibrium of the modeled NC was tested, and then the impacts of different atomic percentages on its mechanical characteristics were explored.

### 3.1. The Results of the Atomic Structure Balancing Step

**Figure 2:**
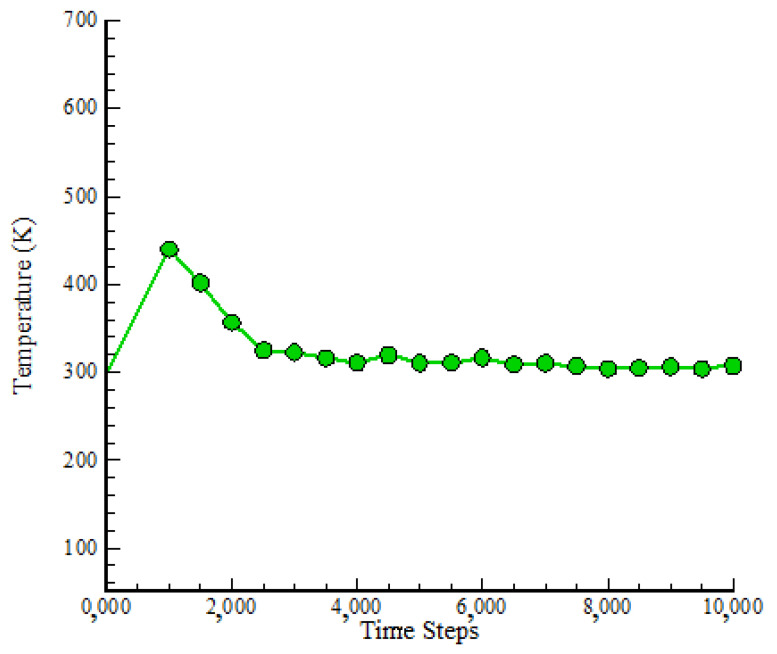
Temp change in simulated atomic samples vs. time step.

The NVT ensemble was utilized to assess two parameters, T and KE, in order to ensure equilibrium. T fluctuations in the simulated NC across 10,000 time steps are shown in Figure 2. According to the findings of the MD simulations, T stabilized at 300 K after 10,000 time steps. This stability was achieved using appropriate modeling parameters, such as T control, pressure attenuation, and interatomic forces. The oscillation amplitude of structures inside the simulated box did not diverge, demonstrating structural stability, as shown by T equilibrium from a physical standpoint. In particular, the final structure attained T equilibrium, and particle mobility was lowered by lowering the oscillation amplitude of each atom provided in the simulation box. The atomic structure’s stability was maintained using appropriate simulation parameters, such as limiting T changes in the MD’s input code. This resulted in atomic samples that were stable. In the equilibration step, a simulation period of 10,000 time steps was found to be sufficient.

**Figure 3:**
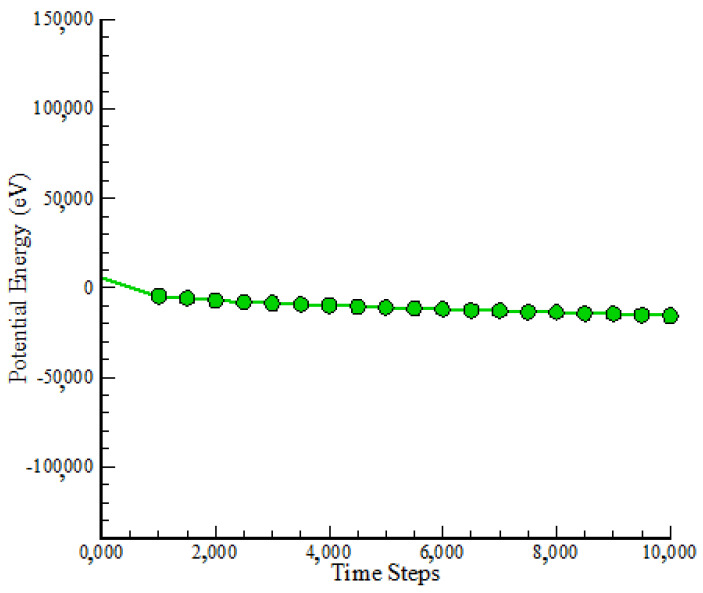
KE change in simulated atomic samples vs. time step.

Figure 3 depicts the variations in PE of the modeled NC over time. It is envisaged that these adjustments would coincide with general T fluctuations. After 10,000 time steps, the numerical value of PE had reached −15,651.15 eV. The convergence of PE with time revealed a reduction in mobility inside the atomic samples. Reducing atom mobility inside the simulation box resulted in fewer oscillations and a more physically stable structure. This equilibrium was approximated by taking into account the attractive forces between distinct components of atomic structure. The proportionality between the specified atomic structure and selected force field was critical in reaching this equilibrium, demonstrating the acceptable approach adopted in this study.

### 3.2. RDF

**Figure 4:**
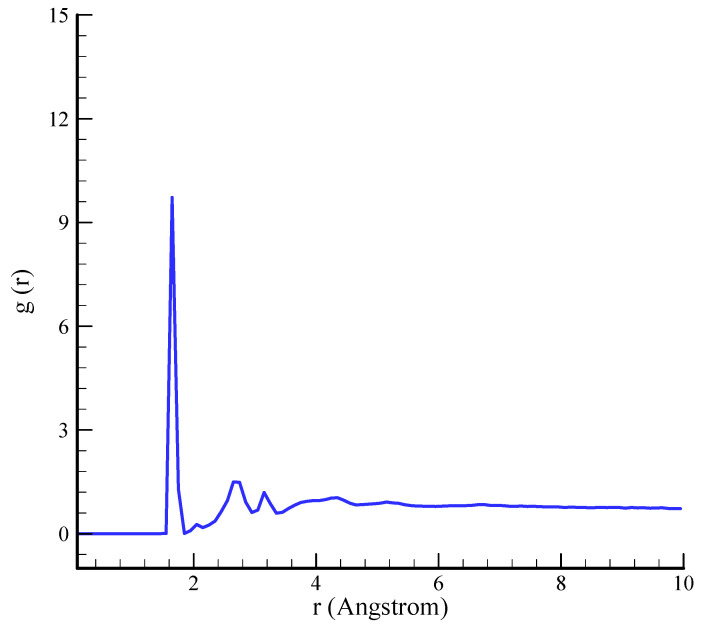
The RDF of simulated silica aerogel sample.

Techniques such as RDF analysis can be employed to conduct a comprehensive examination of the atomic-scale organization and behavior of silica aerogel. This method offers valuable insights into the degree of structural order or disorder, interatomic distances, and atomic coordination within the aerogel. The local atomic environment, which encompasses bond lengths and the material’s overall structural framework, can be more thoroughly comprehended through an examination of RDF profiles. These profiles provide insight into the bonding patterns and arrangement of silicon atoms, thereby revealing the process by which the porous structure is preserved. The RDF diagrams depicted in Figure 4 are consistent with the structures of these materials, which have been previously reported [39].

**Figure 5:**
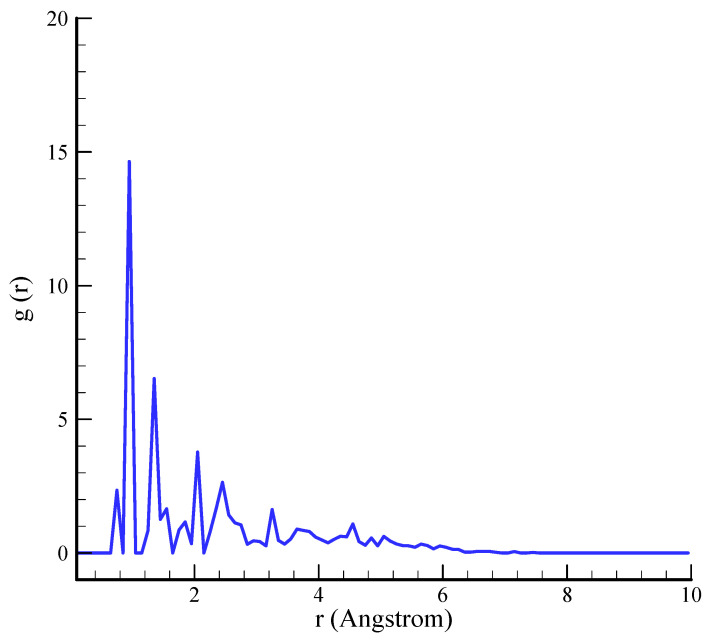
The RDF of simulated chitosan sample.

The atomic-scale structure of chitosan was analyzed using the RDF curve, which was a critical instrument. The local atomic arrangement and interatomic distances within the chitosan network were clearly represented in this diagram, which provided a detailed comparison between the simulation results and laboratory samples. The accuracy of the computational models was verified through RDF analysis, which assessed the degree to which the simulated atomic structure corresponded to the experimentally observed one. The degree of local ordering, atomic coordination, and the structural characteristics of the material at the nanoscale level can be evaluated by analyzing the RDF curve. This method was essential for the validation of simulation predictions and the assurance of the reliability of computational approaches in the investigation of the molecular structure of chitosan. The structures of these materials were consistent with those that were previously reported, as illustrated in Figure 5 [40].

### 3.3. The Results Related to the Mechanical Attributes of the Modeled NC

**Figure 6:**
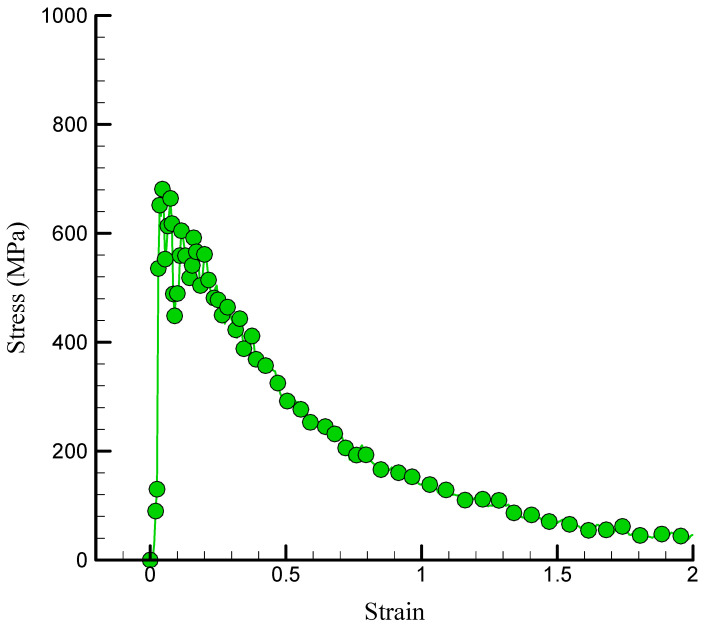
Curve of stress–strain of the simulated NC in the presence of 1% TCP.

This research sought to examine the impact of varying atomic percentages of TCP (2%, 3%, and 5%) on chitosan–silica aerogel nanocomposites by molecular dynamics simulations utilizing LAMMPS software. The investigation was performed at an initial T of 300 K under normal conditions. Figure 6 illustrates the alterations in the stress–strain diagram of the chitosan/silica aerogel/TCP nanocomposite with the inclusion of 1% TCP. The analysis of the stress–strain diagram included an assessment of several parameters to comprehend the mechanical properties of a certain material. The mechanical properties of the targeted nanocomposite were assessed using mechanical testing, revealing that the incorporation of TCP into the silica aerogel nanocomposite improved its tensile strength and toughness. TCP, a biocompatible ceramic material, enhanced the silica aerogel matrix and increased its load-bearing capability. Thus, the stress–strain diagram of NC may demonstrate enhanced stiffness, elevated yield strength, and superior resistance to deformation or fracture relative to pure silica aerogel. The graph’s first linear segment indicated the elastic zone, which was then followed by a deviation from linearity, implying plastic deformation. The apex of the stress–strain curve signifies the ultimate tensile strength, indicating the maximum stress the material can endure prior to failure. A computational investigation indicates that at a strain of 0.04, the US attained 681.145 MPa.

**Figure 7:**
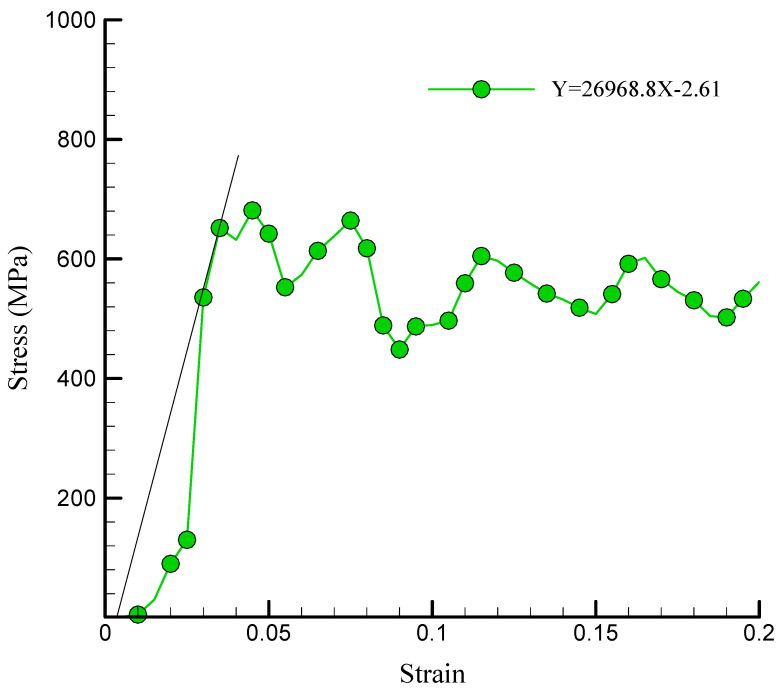
Linear region fitted to the stress-strain diagram (YM) of the simulated NC in the presence of 1% TCP.

The simulated NC’s YM curve in the presence of 1% TCP is depicted in Figure 7. YM is a metric that quantifies the degree of elasticity or stiffness of a material. It represents a material’s capacity to resist deformation when exposed to an applied force and is determined by the stress–strain ratio within the linear elastic range. According to our results, adding TCP to chitosan–silica aerogel NCs enhanced YM. Specifically, after adding 1% TCP, YM reached 26,968.8 MPa. As previously stated, TCP is a ceramic substance noted for its excellent hardness and strength properties. Incorporating this material into NCs resulted in a reinforcing phase, which improved the overall mechanical properties.

**Figure 8:**
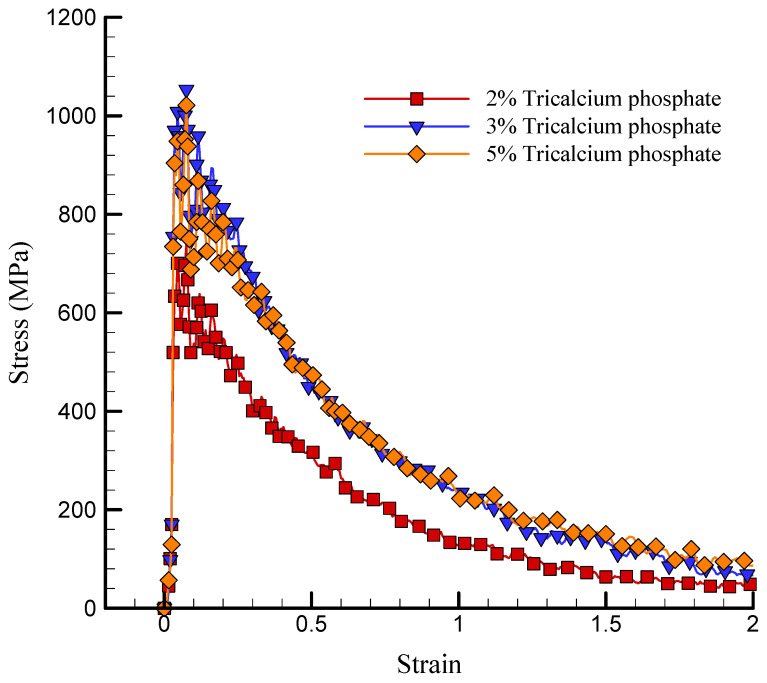
Curve of stress–strain of the simulated NC in the presence of various values of TCP.

The stress–strain curve of the simulated NC in the presence of a variety of TCP values is depicted in Figure 8. TCP NPs functioned as a reinforcing phase in the NC, as indicated by these findings. As a result, the matrix experienced an increase in strength as a result of an increased concentration of reinforcement. The US experienced a numerical increase from 739.742 MPa to 1053.183 MPa as the atomic percentage of TCP increased from 2% to 3%. The increase in the US was attributed to the reinforcement effect of TCP NPs on the nanocomposite material, which was accompanied by an increase in the atomic ratio of TCP NPs. When the atomic ratio of TCP NPs increased, the mechanical properties of the matrix, including strength and rigidity, were enhanced due to a higher concentration of reinforcing elements. However, the US value decreased to 1021.418 MPa as the atomic percentage of these NPs increased to 5%. The construction of NP aggregation can be attributed to this decrease in strength. In general, the interaction between the chitosan–silica aerogel matrix and the TCP NPs may be the physical explanation for this nonlinear behavior. Previous research, such as that conducted by Patil et al. [36] and Murillo et al. [41], showed that the ultimate strength of silica aerogel can fluctuate considerably, spanning from 200 MPa to 1000 MPa. The density of the aerogel was the primary factor influencing this variation, as denser aerogels generally exhibited higher ultimate strengths. Additionally, the analyses conducted in this paper demonstrated a direct correlation between the elastic modulus and the density of the silica aerogel. The reliability of the results was further validated by the consistent observation of this relationship in both experimental data and simulation models. Additionally, the silica aerogel/chitosan nanocomposite’s US values were estimated to be 681–1053 MPa in the study conducted by Alasvandian et al. [42].

**Figure 9:**
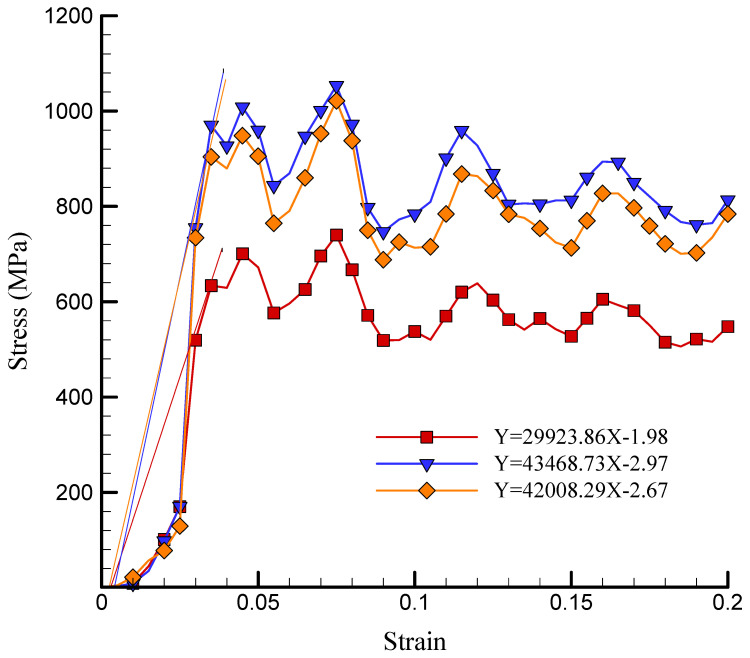
Linear region fitted to the stress-strain diagram (YM) of the simulated NC in the presence of various values of TCP.

The YM diagram of the modeled NC with varying quantities of TCP is depicted in Figure 9. As previously mentioned, the matrix’s surface bond and reinforcement were improved by the addition of TCP to the chitosan–silica aerogel NC, resulting in an increase in strength and rigidity. Additionally, the material’s deformation resistance was improved by the restriction of dislocation movement within the material by TCP NPs. A measure of material hardness, the ratio of stress to strain in the linear portion of the stress–strain curve is referred to as YM. When the atomic proportion of TCP increased from 2% to 3%, the simulated NC’s YM increased from 29,932.86 MPa to 43,468.73 MPa. However, the YM decreased to 42,008.29 MPa when the TCP NPs were further increased to 5%. Incorporating 2% to 3% TCP-NPs into chitosan–silica aerogel NCs increased their YM from a physical perspective by enhancing the interfacial bonding between the NPs and the polymer matrix. This improved stress distribution, load transfer, and resistance to deformation [43]. However, when the TCP content reached 5%, the NPs aggregated, disrupting their uniform dispersion and forming localized stress concentration points. This weakened the material and reduced YM. This behavior is consistent with research on aerogel-based biomaterials, where NP dispersion was a critical factor in determining the mechanical properties [44].

**Figure 10:**
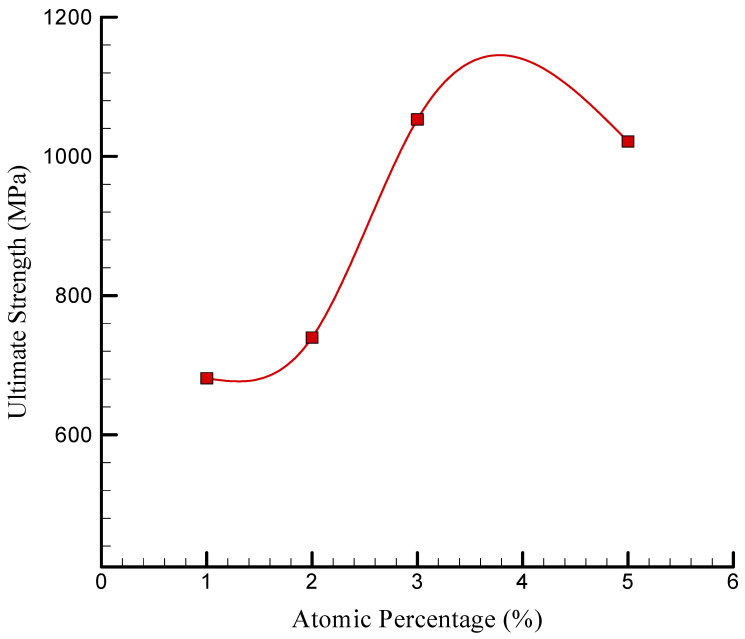
Changes in the US of the simulated NC vs. the atomic percentage of TCP.

The atomic percentage of the US of simulated NC is depicted in Figure 10. The US increased from 681.145 to 1053.183 MPa after the concentration of the TCP NPs was increased from 1% to 3%. This enhancement can be attributed to a variety of factors, such as the fortifying influence of NPs. The atomic percentage of TCP NPs increased, resulting in an improved load transfer and an overall strength enhancement as they dispersed more evenly throughout the NC. Additionally, the surface binding between the matrix and these NPs was enhanced by their addition, resulting in a reduction in separation at the interface and an increase in strength. Nevertheless, the US decreased to a value of 1021.418 MPa when the atomic percentage of NPs increased to 5%. The accumulation of particles and the heterogeneous dispersion that occurred with a higher content of TCP NPs can be attributed to this decrease. In this instance, the mechanical attributes were diminished as a result of the stress concentrators, which were larger particulates and the result of accumulation and inhomogeneity. In fact, the mechanical properties of the polymer NCs were substantially influenced by the behavior of the NPs. The US of the composite can be improved by NPs at lower concentrations as a result of effective reinforcement. However, when the NP content increases, aggregation may develop, resulting in structural flaws and stress concentrations that may impair the composite’s mechanical performance. Nanocomposite research has extensively documented this phenomenon. For example, high reinforcing content often caused particle clusters, resulting in stress concentration areas that weakened the composite structure and diminished tensile strength [45]. Furthermore, NP dispersion was critical; agglomeration reduced the intended composite properties [46]. These characteristics were influenced by interfacial bonding between the NPs and the polymer matrix, as well as the uniformity of NP dispersion. Strong interfacial bonding may expand the interphase area, improving mechanical characteristics, while insufficient dispersion can cause aggregation and reduced performance [47].

**Figure 11:**
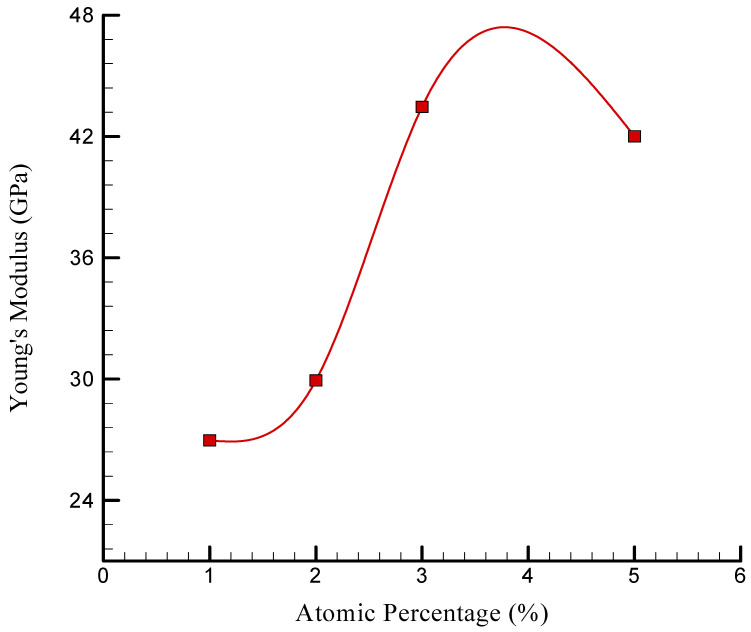
Changes in the YM of simulated NC vs. the atomic percentage of TCP.

Figure 11 depicts the YM of the simulated NC as a function of the atomic proportion of TCP NPs. The numerical findings show that raising the concentration of these NPs from 1% to 3% increased YM from 26.968 to 43.468 GPa. This rise was attributed to their strengthening properties. However, when the atomic proportion hit 5%, the YM dropped to 42.008 GPa. This decline may be due to comparable causes, such as particle accumulation and heterogeneous dispersion, which result in greater stress concentrators. Furthermore, the reduction in YM might be due to the high atomic percentage of TCP NPs and the aggregation phenomena, which prevented the development of strong surface bonds. As a result, the weaker link lowered the charge transfer efficiency between the NPs and the matrix, lowering YM. In conclusion, introducing TCP NPs at a specific concentration increased the NC’s US and YM owing to their strengthening actions and better surface bonding with the matrix. However, surpassing this concentration led to detrimental consequences, such as particle aggregation and heterogeneous dispersion, resulting in lower mechanical characteristics.

Table 2 presents the numerical results concerning various atomic percentages of TCP NPs and their effect on mechanical attributes, such as stress–strain behavior, US, and YM in a chitosan–silica aerogel NC.

## 4. Conclusions

The rigidity and hardness of chitosan–silica aerogel NCs were improved by the presence of TCP, which served as a reinforcing agent. This study examined the prospective applications of chitosan–silica aerogel NCs in bone tissue engineering by examining the effects of varying atomic percentages of TCP (2, 3, and 5%). The results of the MD simulation and analysis with LAMMPS software indicated that T stabilized at 300 K after 10,000 time steps, indicating structural stability and equilibrium within the simulation box. In addition, the PE decreased over time, indicating a reduction in atomic mobility. This resulted in a more balanced structure and fewer oscillations. The PE reached −15,651.15 eV after 10,000 time steps, as measured numerically. The US of the silica aerogel NCs was increased from 681.145 to 1053.183 MPa by the addition of TCP NPs from 1 to 3%. Nevertheless, the US decreased to 1021.418 MPa as a result of particle accumulation and heterogeneous dispersion when the atomic percentage of TCP NPs increased to 5%. In the same vein, YM increased from 26.968 to 43.468 GPa after the concentration of the TCP NPs increased from 1 to 3%, which indicated a strengthening effect. Nevertheless, YM experienced a minor decrease to 42.008 GPa when the atomic percentage of TCP reached 5%. These results suggest that the mechanical properties of the chitosan–silica aerogel NCs were enhanced by the incorporation of TCP. However, there was an optimal range for the atomic percentage of TCP NPs that optimized their strength and rigidity.

## Figures and Tables

**Table 1:** Parameters of Lennard-Jones potential function for particles in MD simulations.

Particle Type	ε (kcal/mol)	σ (Å)
C	0.105	3.851
F	3.0124	2.7329
O	0.06	3.50
H	0.044	2.886
N	0.415	3.995
Si	0.0043	3.69

**Table 2:** Mechanical attributes of chitosan/silica aerogel/TCP NC.

Atomic Percentage of TCP (%)	US (MPa)	YM (GPa)
1	681.145 (±3)	26.968 (±1.02)
2	739.742 (±5)	29.932 (±2.1)
3	1053.183 (±2)	43.468 (±0.89)
5	1021.418 (±4)	42.008 (±1.25)

## Data Availability

All data used during the study appear in the submitted article.

## Abbreviation

Nanocomposites	NCs
Tricalcium phosphate	TCP
Poly-3-hydroxybutyrate	PHB
Temperature	Temp
Amorphous Calcium Phosphate	ACP
Nanoparticles	NPs
Hydroxyapatite	HA
Molecular Dynamics	MD
Potential Energy	PE
Ultimate Strength	US
Young’s Modulus	YM
Radial Distribution Function	RDF
